# A Qualitative Investigation to Underpin the Development of an Electronic Tool to Assess Nutrition Literacy in Australian Adults

**DOI:** 10.3390/nu10020251

**Published:** 2018-02-23

**Authors:** Alyssa M Cassar, Gareth S Denyer, Helen T O’Connor, Janelle A Gifford

**Affiliations:** 1 Faculty of Science, University of Sydney, Camperdown, Sydney, NSW 2006, Australia; amcassar@hotmail.com (A.M.C.); gareth.denyer@sydney.edu.au (C.S.D.); 2 Faculty of Health Sciences, University of Sydney, 75 East St, Lidcombe, Sydney, NSW 2141, Australia; helen.oconnor@sydney.edu.au; 3 Boden Institute of Obesity Nutrition Physical Activity and Eating Disorders, University of Sydney, Camperdown, Sydney, NSW 2006, Australia; 4 Charles Perkins Centre, University of Sydney, Camperdown, Sydney, NSW 2006, Australia

**Keywords:** nutrition literacy, electronic tablet, health literacy, mobile applications, nutrition education, nutrition knowledge

## Abstract

Nutrition literacy is linked to health via its influence on dietary intake. There is a need for a tool to assess nutrition literacy in research and dietetic practice. We sought guidance from nutrition professionals on topic areas and features of an electronic nutrition literacy assessment tool for Australian adults. 28 experienced nutrition professionals engaged in a range of nutrition and dietetic work areas participated in six focus groups using a semi-structured interview schedule. Data were analysed using an inductive approach using NVivo 10 (QSR International, Pty Ltd., Doncaster, Australia, 2012). Key areas identified to assess nutrition literacy included specific nutrients versus foods, labels and packaging, construction of the diet, knowledge of the Australian Dietary Guidelines and Australian Guide to Healthy Eating, understanding of serve and portion sizes, ability to select healthier foods, and demographics such as belief systems and culture. Exploitation of electronic features to enhance visual and auditory displays, including interactive animations such as “drag and drop” and virtual reality situations, were discussed. This study provided insight into the most relevant topic areas and presentation format to assess the nutrition literacy of adult Australians. The visual, auditory, and interactive capacity of the available technology could enhance the assessment of nutrition literacy.

## 1. Introduction

Understanding health and nutrition information requires adequate literacy and numeracy skills, which are collectively referred to as health literacy [1]. The World Health Organisation defines health literacy as the “cognitive and social skills which determine the motivation and ability of individuals to gain access to, understand and use information in ways to promote and maintain good health” [2]. Health literacy encompasses many factors, including the knowledge and skills required to understand and apply information relating to disease prevention, first aid, safety, accident and emergency procedures, drug and alcohol risks, and lifestyle factors supporting maintenance of good health status [3], and may be a more important determinant of a person’s health than age, income, employment status, education level, and culture [4].

Health literacy is associated with health behaviours and outcomes [5,6,7,8]. Individuals with poor health literacy skills may have a 1.5–3 fold increased probability of experiencing an adverse health outcome [6], potentially due to an inadequate understanding of the ways in which related symptoms can be reduced or treated [4]. Lower levels of health literacy have been associated with greater hospitalisation rates, reduced ability to comprehend labels and health messages, less awareness about their own condition or disease, decreased overall health status, and higher risk of death [6,7]. Data from the Australian Bureau of Statistics indicate that only 43% of the Australian-born adult population has adequate levels of health literacy [3]. From a public health perspective, health literacy is considered an asset to be built, as a product of health education and communication that encourages greater empowerment in health decision making [9].

Nutrition literacy is analogous to health literacy, as it is defined by the ability of individuals to obtain, process, and understand basic nutrition information and services they require to make suitable dietary selections [10]. Sound health and nutrition literacy requires an individual to comprehend health and nutrition concepts and to possess simple numeracy skills. Without these skills, difficulty may be experienced with understanding the concept of a healthy diet, reading nutrition information, determining portion sizes, recognising healthier food choices, and utilising nutrition information sources [1,11,12]. Consequently, these behaviours link to a higher susceptibility to excess weight gain and associated health conditions [13]. Being able to understand what a healthy diet encompasses is considered difficult and may require more advanced cognitive, literacy, and numeracy skills. Ideally, dietitians would adjust their use of educational methods depending on needs and level of nutrition literacy [14].

There is a need for a more specific tool to evaluate nutrition literacy at a population and individual level and to measure the impact of activities, interventions, and policies that are in place to promote healthy eating. A range of tools has been developed to measure health literacy [5]; however, reviews of existing instruments that include some aspect of nutrition literacy indicate that access to valid and reliable assessment of this construct is limited [4,15]. Gibbs and colleagues have recently developed a nutrition literacy instrument based on literature reviews and interviews conducted with eight nutrition professionals from four areas of practice (research, education, outreach, and nutrition education) [13,16,17]. The instrument has been tested for content validity among a broader sample of dietitians, piloted in a small sample of clients [17], and revised and validated in several groups [18,19,20,21]. The focus of this instrument, which has had limited use in electronic form, has become the assessment of nutrition literacy in individuals with chronic disease in a clinical context. It has recently been suggested that work beyond the clinical context is warranted [22]. Guttersrud and colleagues have also reported on the validation of a paper and pencil Critical Nutrition Instrument (CNL), which consists of two scales: “engagement in dietary habits” and “taking a critical stance towards nutrition claims and their sources” [23,24]. While the first scale has been tested for validity in 14–15 year old Norwegian students [24], the instrument may not be suitable for people with lower reading skills, and does not assess across a broad range of nutrition literacy domains. Additionally, critical nutrition literacy is only one component of the broader nutrition literacy concept [25].

Most of the other existing instruments designed to assess general nutrition knowledge or literacy in adults have minimal validation, are now dated, and are not specific to Australian foods or culture. There have been few new tools developed since 2007, with most of these adaptations from previous instruments. One of the key limitations of existing tools is that the readability and comprehension level required to complete the questions has not been undertaken; lower scores on existing nutrition literacy tools may reflect a lower reading level rather than poor nutrition knowledge. The average reading ability of the Australian population (not including migrants educated in non-English speaking countries) is approximately equivalent to a year 8 level (or 13 years of age) [26], and nearly half of all Australians aged 15–74 years possess literacy and numeracy skills below that deemed to be needed to meet the demands of our “knowledge society” [27]. Use of a multimedia device to administer the questionnaire may overcome this barrier [28]. Electronic mediums can capitalize on the use of alternatives to text, such as pictures, audio, movies, or animations that may assist those with lower literacy levels or who do not primarily speak the language used in text-based questionnaires [29].

The aim of this study was to obtain the guidance of dietetic professionals from a larger range of work settings on key nutrition areas that should be included in an electronic nutrition literacy assessment tool (e-NutLit) specifically for Australian adults. Nutrition literacy in this context was limited to the knowledge and skill components required for the selection of a healthy diet for everyday life, rather than an assessment of knowledge or skills in cooking, budgeting, or knowledge of where foods come from. The study also aimed to capture opinion on how to present these item areas within the electronic tool.

## 2. Materials and Methods

### 2.1. Participants

Participants were purposively selected by the research team based on their expertise and experience in a range of work areas in nutrition and dietetics and snowball sampling [30]. A target sample of 38 potential participants were initially contacted by one researcher (H.T.O.) via email with a formal invitation to participate. Invitees could forward the e-mail to other dietitian professionals. Interested study participants were sent an information sheet and consent form by e-mail; anonymity and confidentiality were assured. Participants agreeing to take part spanned all nutrition and dietetics work areas, indicating the end of recruitment for the study. Those participating were polled for available times via Doodle (www.doodle.com) or asked for broad availability (days/times) to be supplied by e-mail.

### 2.2. Procedure

Participants attended one of six focus groups (one face-to-face on site, and five via teleconference). Each group consisted of 4–6 participants and ran for a duration of approximately 60–90 min. Experienced/trained facilitators (J.A.G., H.T.O.) who were also Accredited Practising Dietitians led all sessions. The focus groups were conducted using a semi-structured interview schedule asking participants to describe their experience working with individuals using existing nutrition guidelines (Australian Dietary Guidelines (ADG), Australian Guide to Healthy Eating (AGHE)) [31]; the main guiding questions are provided in Table 1. The interview also probed the communication of nutrition information panels and guidelines to individuals, features of an electronic tool that would be helpful in facilitating the evaluation of guidelines knowledge, and core areas to assess in a nutrition literacy assessment tool. The population guidelines were chosen as a basis for discussion on nutrition literacy, because these are benchmarks of the nutrition quality of an individual′s diet and are used in communicating nutrition and dietary aims; the elements of these guidelines and relative ease in communication would inform the development of a nutrition literacy tool. To “break the ice”, an introduction was given to define the scope of the project. At that time, participants confirmed their areas of work and experience.

Focus groups were recorded and then transcribed by either a project investigator (one focus group was transcribed verbatim by (A.M.C.) or a professional transcription service). Transcripts were de-identified by assigning pseudonyms for each participant and removing identifying information such as place of work. These were returned to participants for verification and correction (within two weeks). Participants were instructed to amend the text to ensure the transcription correctly summarised the content of their specific group discussion. All comments and corrections returned by participants for each focus group were compiled in to a single document for analysis. All focus groups were conducted prior to thematic analysis due to time restrictions within the university research semester for data collection personnel. Past and present work areas identified by participants at the beginning of the interview were classified according to defined Dietetics Association of Australia (DAA) work areas [32].

### 2.3. Data Analysis

Data analysis was conducted using a general inductive analysis procedure [33]. Analysis was initially conducted by one researcher (A.M.C.) and discussed with co-investigators (J.A.G., H.T.O.), and revisions were checked with co-investigators (J.A.G., H.T.O.). Analysis was overseen and finalised by a lead investigator (J.A.G.). Identification of themes that recurred within and across focus groups were achieved using NVivo 10 qualitative data analysis software (QSR International Pty Ltd., 2012, Melbourne, Victoria, Australia). An audit trail of changes to coding was kept. Coding tree hierarchies within the analysis software were used to assist in theme identification and refinement including reduction of overlap and redundancy. After agreement was reached between investigators on major themes, a consensus document was compiled and circulated to the participants to critically review themes and ideas that had arisen across all groups and to consolidate the consensus.

Ethical approval was granted by the University of Sydney Human Ethics Committee (Project No. 2013/633). All participants provided informed consent prior to participation.

## 3. Results

Twenty-eight (74%) of the 38 invited professionals agreed to participate. Five (13%) declined (reasons included: maternity leave, family issues, too busy, no reason provided), and the remaining five invitations (13%) failed to elicit a response. Participants were recruited from all DAA work areas (Table 2) [32].

### 3.1. Nutrition Literacy Assessment Areas

**Figure 1:**
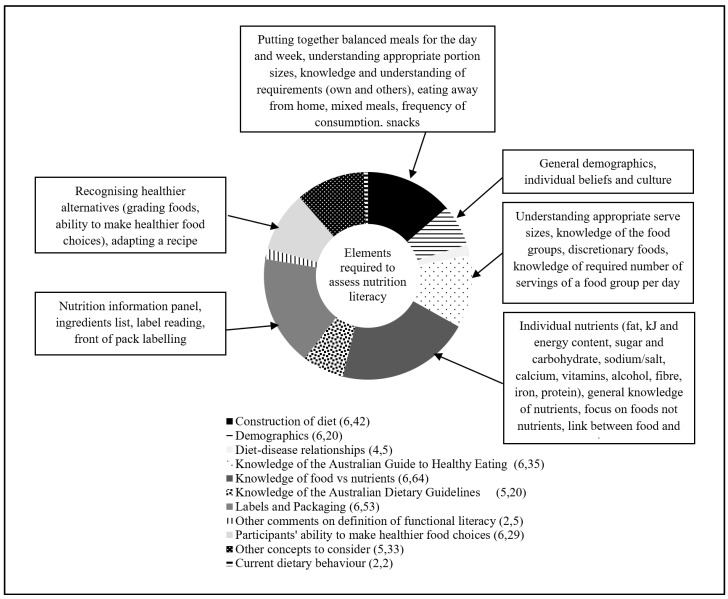
References to elements required to address nutrition literacy in Australian adults. Numbers in parentheses represent the number of focus groups referencing the theme followed by the total number of text references for the theme.

There were six main themes discussed by participants for inclusion in a tool to assess nutrition literacy. Diet-disease relationships were also discussed but did not emerge as a major theme. Exemplar quotes for each theme are provided in Table 3, and an illustration of the density of discussion for themes is provided in Figure 1.

#### 3.1.1. Knowledge of Nutrients versus Foods

Discussion about assessment of knowledge of nutrients versus whole foods was evident across all focus groups. Although there has been a recent emphasis on considering foods rather than nutrients in making recommendations [34], some participants still indicated that testing the nutrient knowledge of Australian adults would be important to include in a tool assessing nutrition literacy. Participants identified specific nutrients (such as salt, fat, and fibre) in their discussion within this theme as examples.

#### 3.1.2. Knowledge of the AGHE and ADG

Participants indicated that knowledge of the AGHE should be assessed, with emphasis placed on assessing the understanding of appropriate serve sizes, basic food groups, and discretionary foods. Participants also discussed addressing knowledge and understanding of the ADG with the recommendation to assess general awareness of the ADG and the messages they convey.

#### 3.1.3. Food Packaging

Testing the respondents’ ability to read, understand, and interpret food packaging was a predominant theme across all focus groups. Assessment of Nutrition Information Panels (NIP) drew a substantial amount of this discussion across the groups. Participants also commented that the ingredients list should also be featured in an e-NutLit and indicated that it would be important to evaluate awareness of the ingredients list, its purpose, and if it influenced their food choices. The importance of determining if there were specific populations more likely to use ingredient lists was also raised.

#### 3.1.4. Construction of a Healthy Diet

The ability to construct a healthy diet was nominated as a key area to include in the e-NutLit, particularly regarding skills in selecting a balanced intake (including individual meals, days, and a weekly intake) and skills in selecting appropriate portion sizes.

#### 3.1.5. Making Healthier Food Choices

Discussion pertaining to assessment of skills in making healthier food choices focused predominantly on recognition of healthier foods, including selection of these within each food group (for example grading the foods) and adapting recipes. Grading foods as a method of assessing whether the individual can select or rank foods that are healthier and more consistent with dietary guidelines was a common subtheme.

#### 3.1.6. Demographic Characteristics

Although this is not an area that can be “assessed” as such, participants considered these an important inclusion an instrument to assess nutrition literacy. Along with general descriptors (age, sex, social economic status, etc.), cultural and individual beliefs (e.g., religion, nationality, and cultural beliefs/taboos including vegetarianism) expected to have a strong influence on nutrition literacy were discussed.

### 3.2. Electronic Tool Presentation Features

Three main categories related to electronic tool presentation features were identified: visual representation, audio, and other (miscellaneous) features. Discussion on audio did not feature heavily, but was discussed as an option to assist participants to recognise images or words, for those with visual impairments or for specific languages. Other general suggestions were in relation to cultural considerations, order of presentation, and consideration of industry influence (packaging) on perception of food images. Exemplar quotes for each theme are given in Table 4. Within visual representation, four major specific themes emerged.

#### 3.2.1. Interactive Animations

When probed with potential animation features, “drag and drop” was discussed positively with supporting for its specific suitability in lower literacy groups. Other potentially valuable interactive features included virtual reality situations (e.g., food hall) and expand and fill images.

#### 3.2.2. Presentation of Mixed Foods

This theme included subcategories: relationship of AGHE serves to everyday food presentation and single versus multi-ingredient or prepared foods. There was substantial cross-over between talk on the relationship of AGHE serves to everyday food presentation and talk on presentation of single versus multi-ingredient or prepared foods; the abundance of mixed meals or pre-prepared foods in everyday life and the potential need to identify serves or ingredients for the tool was frequently discussed concurrently.

#### 3.2.3. Practical Suggestions on Item Presentation

This category included the idea of building meals and recipes or diets, having multiple choice questions, and using pictures instead of words.

#### 3.2.4. Food Images

Aspects of food images were discussed, including the use of common items for scale (e.g., utensils), ensuring accurate and realistic portion sizes in images, and using realistic and clear food images.

## 4. Discussion

This study identified key elements for inclusion in an electronic nutrition literacy assessment tool for adults specific to the Australian context and informed by nutrition professionals from a broader range of work settings than that of previous work. Several main elements were identified within the context of assessing competency in making everyday decisions regarding selection of a healthy diet. Recommendations for the design features of the tool strongly supported use of visual images of real foods, fiduciary markers to indicate portion size, interactive components such as ‘drag and drop’, and the potential capacity for auditory item stems.

One of the key deliberations of the focus groups revolved around the benefits of assessing knowledge of nutrients versus knowledge of foods. There was support for assessing key nutrients (e.g., salt, fat, fibre) and energy, as well as knowledge of food groups and healthier food choices. The healthfulness of food choice was also referenced with specific nutrients (e.g., choosing foods lower in salt or sugar). Gibbs et al. suggest that greater knowledge of some nutrients may be important for people with particular conditions such as diabetes [13,16]. Although there is a large body of research that focuses on the effects of isolated nutrients on health and disease, there is increasing recognition of the biological complexity of foods and health [35], and of the benefits of addressing eating patterns and dietary quality. In fact, there has been a recent shift away from nutrient-based recommendations to considering whole foods in the context of dietary patterns [34]. The latest release of the ADG makes recommendations based on whole foods on the premise that most people following the guidelines would meet nutrient recommendations and that advice on single nutrients is difficult to apply [31]. Knowledge of dietary guidelines may lead to healthier food choices in the absence of more technical information [36]. However, the ADG refer to nutrients; they give examples of food choices and recommend that people read labels to choose lower sodium options [31]. Thus, the theme of knowledge of foods and nutrients feeds into the themes discussing the assessment of knowledge of the ADG and the AGHE, and supports assessment of both ‘foods’ and ‘nutrients’ in a nutrition literacy tool for adults.

Participants favoured assessment of ADG concepts rather than having knowledge of its wording. Together with knowledge of the AGHE, assessment of understanding appropriate serve sizes, knowledge of the food groups, and discretionary foods were discussed most. This confirms the work of Gibbs et al. who suggest that competence with household measurements and understanding good groups are important nutrition literacy skills [13,16]. All groups discussed assessment of serve sizes and some included reference to “portion size”. Individuals may have difficulty estimating serve sizes compared to national guidelines [37]; some foods eaten by Australians are 30–160% larger than AGHE serves while others are 30–90% smaller (for dairy, some fruits, and non-starchy vegetables) [38]. Foods may sometimes be eaten at meals in larger portions than one standard AGHE serve (e.g., bread in a sandwich); however, typical portion sizes for discretionary foods can be up to 400% [38] of a standard serve when the energy budget of many people does not allow for any serves from this group [31]. Understanding of recommended serve sizes also plays a central role in consuming a balanced diet for good health, because consuming the optimum serve size and number provides the required intake of vitamins, mineral, and macronutrients over time [31]. Assessment of the ability to estimate AGHE serve sizes, knowledge of food groups, and the number of recommended serves in an e-NutLit may provide further insight into health and disease in selected groups.

Other major themes identified discussed assessment of interpretive or functional nutrition literacy rather than knowledge. Labels and packaging were discussed by all groups. Many references to labelling were about the NIP; participants generally supported the assessment of participants’ ability to read an NIP in an e-NutLit, particularly interpreting information about specific nutrients, and being able to make an informed choice. The ingredient list was also favoured as an item for the assessment of nutrition literacy. Being able to apply numeracy and literacy skills to reading and comparing labels may be important in making healthier food choices [12,39]. This may be particularly important in vulnerable populations, as they are at greater risk for nutrition-related conditions [40]. Evidence suggests that food labels and packaging are often misunderstood by those with a lower socio-economic status, and an inability to interpret food labels is highly correlated with low literacy and numeracy levels [41,42]. However, those with higher literacy levels can also experience difficulty with food label or package interpretation [42]. We recently piloted an electronic food label literacy tool that demonstrated that although a higher level of health literacy was strongly associated with label reading literacy, those with higher health literacy still scored poorly in some areas, particularly on calculations [43].

The ability to differentiate between an AGHE serve, serving size on a package, and portion size at an eating occasion may all present difficulties to the average consumer. A major challenge for the general population in reading food labels/packaging appears to be interpretation and application of the serve size on the label; this can lead to either an over- or under- estimation of the amount of nutrients consumed [42]. Nutrition-related claims are also reported to be confusing for many consumers. Statements such as “no added sugar” are often interpreted as the product containing no sugar [41]. Such nutrition claims have been suggested to encourage increased consumption of products that are perceived as healthier [41]. However, food label use has been associated with improved dietary quality [44]; therefore, the assessment of the ability to correctly interpret food labelling information would be an important aspect of nutrition literacy.

The need for nutrition literacy assessment instruments to support a greater range of literacy levels evident in the Australian population suggests the potential benefit of using an electronic platform to assess nutrition literacy. Use of pictures when communicating health information can provide significant benefits to attention, comprehension recall, and intention/adherence [45]. Simple drawings and photographs, especially for low-literacy populations, can assist in understanding the intended message [45,46], while technology such as touchscreens can also facilitate survey administration in this group [47,48]. There is also the potential to include spoken question stems. To our knowledge, no contemporary electronic tool exits to measure nutrition literacy.

Innovative question types that incorporate the interactive drag and drop feature, supported by the participants in the current study, and problem-solving simulations also allow examination of the respondents’ knowledge, skills, and capabilities [49]. Virtual reality situations were discussed as an option in some of the focus groups. Those operating the tool would be exposed to life-like scenarios that would enable them to navigate around environments such as food halls that would have a variety of takeaway and fast food options available, or a supermarket where they would be required to select items. This type of technology has already been used in marketing and retail environments, and has also been tested amongst university students with positive results [50]. Virtual reality scenarios are a concept that holds a great deal of potential in health research, and may be developed for use in populations with low literacy [51].

References were made to visual representation of accurate and realistic images of food and portion sizes. Indication of scale was also raised as a consideration when presenting images in this tool. Examples given included comparing a piece of meat to the size of a hand or using a fork near a plate to show the size of the plate to allow participants to gain a better understanding of the serve or portion size being displayed in the image. Respondents may be better able to respond to questions on portion size by ensuring that a standard reference is included [52]. Measurement aids are commonly used to enhance the accuracy of portion size estimates when weights are not practical [51].

Audio or language features may facilitate utility across a larger number of respondents including those with English as a second language or those with lower levels of literacy. Hahn et al. (2004) developed a ‘Talking Touchscreen’ to accommodate varying literacy levels and computer skills to enable cancer patients to complete a quality of life assessment. The tool was developed so that one item was presented at a time on the computer touchscreen, supported by a recording of the question [47]. Various colours, text styles, and graphic images were used to increase visibility, and a small icon appeared near the text to allow the audio to be replayed as required [47]. This tool was demonstrated to be effective for ethnically diverse populations with varying literacy abilities, education levels, and computer skills. They indicated the tool was easy to use and comprehend, and commented on the practicality of the multimedia approach [47].

The main strength of this study was recruitment of a diverse range of expert dietetic professionals from all DAA work areas. The inclusion of input from a broader range of nutrition work areas (e.g., food service and industry) supports assessment of nutrition literacy in both clinical and non-clinical contexts as suggested by Velardo [22]. It also contributed to a comprehensive scope of opinions and nutrition assessment areas that are going to be addressed in an e-NutLit, as well as input on presentation techniques via an electronic format, which may be especially useful for lower literacy populations [28,29,47]. Sourcing input from Australian dietitians allowed a specific focus on an instrument for the Australian context. Using focus groups via telephone facilitated the collection of a rich and diverse discussion, as each participant’s opinions were refined by others. A level of anonymity is provided by the telephone format, as well as being able to access participants from a range of geographic locations in a time-flexible way. Individual and face-to-face interviews may not have yielded the same richness in discussion or number of participants. De-identified transcripts were emailed back to participants for approval and further comment, and this additionally afforded each participant the opportunity and time to deliberate on the focus group discussion and make any further individual comments that may have been forgotten or omitted due to time constraints. This method also enhanced the rigor of the results, as it gave participants the opportunity to correct what may have been transcribed incorrectly or portrayed in conversation inadequately. A limitation of the study was the non-representative sample of dietetic professionals, possibly affecting the range of data collected. Additionally, since few of the participants had experience with the existing nutrition knowledge/literacy assessment tools, electronic surveys, or application development for smartphones/tablet personal computers, their expertise in this realm was not extensive.

## 5. Conclusions

This study gathered valuable insights from focus groups of experienced nutrition professionals regarding the assessment of nutrition literacy (specifically, nutrition knowledge and food selection skills). Key areas identified by participants included knowledge of specific nutrients and foods, knowledge of the ADG and AGHE, appropriate serve and portion sizes, reading and interpreting food labels and packaging, and the ability to select healthier foods. Existing nutrition literacy assessment tools have minimal validation, are dated, and are not specific to Australian culture and foods. Many do not use visual aids and only incorporate some of these themes. Using currently available technology should enhance the assessment and usability of a nutrition literacy instrument, especially for low-literacy groups. This study will inform items and a format for an e-NutLit suitable for adult Australians.

## Figures and Tables

**Table 1:** Guiding questions for group discussion on development of an electronic tool to assess nutrition literacy.

Guiding Question
Could you describe your experience working with individuals using nutrition guidelines?
What problems have you encountered with communicating the Australian Dietary Guidelines to individuals?
What problems have you encountered with communicating the Australian Guide to Healthy Eating?
What problems have you encountered with communicating nutrition information panels?
What have you found helpful in communicating these nutrition guidelines to individuals?
If an electronic resource were available, what features would it have to facilitate evaluating guidelines knowledge of individuals?
What do you see as core to assess in such a tool?

**Table 2:** Number of participants in Dietetics Association of Australia work areas.

Dietitians Association of Australia Work Areas	Number of Participants
Hospital */nursing home	13
Food service	4
Community health	9
Government department	3
Educational institution **	12
Private practice	6
Industry	8
Public health nutrition ^†^	10
Other ^‡^	11

* Includes any mention of “clinical” work; ** Educational institution also includes PhD/research; ^†^ Added Public Health Nutrition to Dietitians Association of Australia Work Areas; ^‡^ Includes media/communications, and one student (not graduated).

**Table 3:** Example quotations on areas to assess nutrition literacy.

Theme	Code	Number of References ^†^	Quotation ^‡^
Knowledge of nutrients versus food			
	General knowledge of nutrients	11	Just on the point of linking foods with nutrients I think that might be maybe a key activity having the three main or like carbohydrate, protein, fat, vitamins and minerals as an option with a list of food pictures and just ticking or selecting the options of which that food might relate to that was an activity that we’ve worked—has worked well with very low literacy groups just linking the actual role of food to the food type. (Wanda, FG5)
	Focusing on foods, not nutrients	4	I think a whole food approach and maybe even a meal approach (Dianne, FG5)
	Fat	14	The two areas that you might want to consider I think would be energy content and saturated fat. I think trying to disentangle saturated fat from total fat would be useful. That the focus is on saturated fat and not total fat. (Wilson, FG4)
	Energy content/kJ (kilojoules)	10	I still think energy content of meals and choices is a really key thing that needs to be looked at. Can people identify which is a lower kilojoule option when they’re presented with several food options in front of them? (Wilson, FG4)
	Sugar and carbohydrates	8	With the nutrient side of things it’s also an important message to talk about added and natural sugars because that’s something at the moment, everyone’s just—sugar is getting a bad name for everything, so I do think they need to be used in conjunction and maybe looking at it more from a whole food point of view rather than reducing the intake of refined foods and for these reasons of them either higher in sugar, salt or/and fat. (Rachel, FG 6)
	Sodium or salt	5	Australians need to reduce their salt intake and then go for questions as to how you do it. You could even say ‘Australians need to reduce their salt intake because half the population has high blood pressure by the time they’re fifty or whatever and this costs us x billion dollars per year for the medication’. I don’t know if I’d go that far but just to give a rationale that the medical evidence suggest that most people need to reduce their sodium intake from salt and then you look at how you might do it. (Sonia, FG4)
	Calcium	4	Parents had forgotten that you need calcium for children so therefore they need dairy food. (Georgia, FG6)
	Vitamins	2	They had forgotten that vitamin C is an essential vitamin and therefore you need citrus fruits. I think they do need to have some understanding of the vitamins and the minerals. (Georgia, FG6)
	Alcohol	2	Maybe knowing how much alcohol is recommended, what the actual guideline is for alcohol (laughter). Even though it changes, probably, yeah, knowing what it’s supposed to be so you know what to limit down to. (Hayley, FG1)
	Fibre	1	I don’t think it’s wrong to have some nutrient focused questions if we’re talking about nutrients like sodium, saturated fat but also fibre, calcium and iron potentially and then seeking knowledge about which foods are the best sources of these nutrients. (Wilson, FG4)
	Iron	1	I don’t think it’s wrong to have some nutrient focused questions if we’re talking about nutrients like sodium, saturated fat but also fibre, calcium and iron potentially and then seeking knowledge about which foods are the best sources of these nutrients. (Wilson, FG4)
Knowledge of the AGHE and Australian Dietary Guidelines			
	Understanding appropriate AGHE serve sizes	12	I certainly think that serving sizes and the number of recommended serves is a key part that have to be, I would imagine a part of any sort of tool (Wilson, FG4)
	Knowledge of the food groups (AGHE)	11	Have the groups already up there with different foods under it and getting people to select them into the appropriate group some people think that ice-cream comes under the dairy category. (Rachel, FG6)
	Discretionary foods (AGHE)	9	It’s funny what some people think is healthy. Yeah, I just think that even things like fruit juices where they might get them from Boost Juice^®^ and they’re thinking that that’s one serve of fruit, but then again that will still fall under that fruit category, well technically juice does, but if there’s a whole heap of sugar and stuff added to it is that then technically a discretionary food? I think there are those sorts of things where you might find, yeah, that that affects what they consider a healthy diet to be. (Rachel, FG6)
	Knowledge of required number of serves from a food group per day (AGHE)	3	So I think obviously having a section on core food groups pick how many pieces of fruit you should have a day, or the minimum amount of dairy. (Sally, FG2)
	Knowledge of the Australian Dietary Guidelines	20	They actually specify fat, sugar and salt and I suppose if you measured people’s ability to determine which of two foods or which of three foods have different levels of those nutrients I suppose what it would indicate is how good the Dietary Guidelines might be in terms of changing people’s knowledge. I guess the next question is, “Does that make any difference to consumption?” (Bridget, FG6)
Food Packaging			
	Nutrition information panels	35	What conclusions should they be drawing from the panel, and what’s meaningful to them, and how to use it is probably the most helpful thing. (Sarah, FG3)
	Ingredients list	11	It would be interesting to find out what people’s perception is of the ingredient list, because I was just, you know well participant said ‘there’s more information than just the energy and macronutrients but with the ingredient list, I’d be interested to know whether people look at something like this one, for example, banana 8%, strawberry 6%, do they go “well that’s great, it’s got fruit in it, obviously that fits in to my healthy eating, my food groups so I’m going to consume that”. That would be interesting to sort of look at that level, their perception of the ingredients. (Patricia, FG2)
	Label reading	4	But maybe in addition there should be something about skills in, say, label reading. I mean we have labels and we have lots of nutrition information, we want people to be able to use it in a different way. So I would personally like to see that as part of any sort of nutrition literacy tool. (Wilson, FG4)
	Front of pack labelling	3	So I think people need to look at that ingredient list and hopefully front of pack labelling will give them a bit more quick information when they’re actually shopping—although it’s also intended to be something people observe when they’re sitting at the breakfast table, reading the cereal or milk and the information on it. (Sonia, FG4)
Construction of diet			
	Putting together balanced meals for the day/week	10	You’d have a protein in a section and fruit or veggies in another and grains in another bit and so you’d sort of be able to, that works for a meal rather than a day, but I think that idea of being able to build, build your meal or build a day. (Bec, FG2)
	Understanding appropriate portion sizes	10	I guess we’re recommending they need to just have an understanding of the right portion size for their needs, or for the needs of the people that they are catering for. (Danielle, FG3)
	Knowledge and understanding of own and others requirements	7	If somebody’s exercising a lot, if somebody has a certain disease, if somebody’s caring for somebody else, if somebody’s overweight, maybe just can they prepare a meal a day of food choice for certain people as well. (Heidi, FG5)
	Eating away from home	6	You have to remember that labelled food probably makes up at most two thirds of what people eat, probably less than that. And so we don’t want to forget all of those out of home occasions when people are buying food too. So in a way we want to test their skills to be able to make those judgments without reading labels. (Wilson, FG4)
	Mixed meals	4	It seems to be one area where people just throw their hands up and forget about the serves because you can’t pull out every single ingredient or it may be above their level to be able to pull out every ingredient and stick it into a food category and work out how much their getting in that meal or in a day. So that would be a key example I think. (Wanda, FG5)
	Frequency of consumption	3	Obviously how often you have a food is as important as how much there is there. (Ben, FG5)
	Snacks	2	I think snacks and what people consider as a snack in the first place, but picking out and choosing healthier options in lists of snacks would be another area to look at as well. (Wendy, FG6)
Participants ability to make healthier food choices			
	Recognising healthier alternatives	27	I think two things that are relevant to all people, irrespective of their energy requirements, are the need to eat a variety of food, and to make healthy choices within those food groups, and that’s applicable to all. But I think that fundamental concept of so many groups of foods, healthy choices within, you can apply that universally. (Sam, FG3)
	Adapting a recipe	2	Or you could do something specific like how would you increase the fibre in this meal or reduce the saturated fat in this meal? (Wendy, FG6)
Demographics			
	General (Age, sex, education level, lifestyle factors, diets followed)	10	So whenever you do any sort of survey the demographics of that group is really important. (Irene, FG5)
	Knowledge of the individual’s beliefs and culture	10	Yeah from the point of view of setting up the literacy tool, you probably have to look at peoples’ source of belief in working with individuals. I mean who have they believed and why? But looking at their belief system I think is probably the most important thing if you’re going to get through to an individual. (Sonia, FG4)

^†^ Number of references = the number of times a related theme was coded in NVivo. ^‡^ Names presented with the quotations are pseudonyms, and focus group number (FG1–FG6) is indicated. AGHE: Australian Guide to Healthy Eating.

**Table 4:** Example quotations on presentation aspects of an electronic nutrition literacy tool.

Theme	Code	Number of References ^†^	Quotation ^‡^
Visual representation			
	Interactive animations	30	So the drag and drop concept is an interesting one, provided that—two things: one that the drag and drop components were in meaningful portions of the way people eat it, or prepare it. So for example, is this a half a mango if they’re going to have a serve, or how much meat, or the vegetables, or the pasta. And then for the second part, being that if we want to enable them or increase their knowledge, is that within any group, or what you’re serving, is there a good, better, best. And if I choose this meat verses that meat, understanding the impact of that, and then being able to see it, on what that would do for their nutritional status. (Sarah, FG3)
	Presentation of mixed foods ^§^	30	And I think more broadly, part of the issue is that when it comes to nutrition guidelines or Australian Guide to Healthy Eating or dietetic advice in general, a lot of it is based around assembling meals from scratch, like one would do in one’s kitchen. But a lot of people’s experience with food is like at a food hall, where they go when meals are already prepared for them. So the advice that they might have been given and how to apply it, is just met with a huge disconnect, so it’s not simple. (Sam, FG3)
	Practical suggestions on presentation and assessment	22	The other thing you might want to look into (name) is the whole area of infographics, so the style of the way—it could still be a multiple choice question but you can say a lot more of the question with a visual infographic which is asking the question not just the responses as well. Very clever infographic designers these days can turn one very long, long question into a quick infographic that poses the same thing. (Samantha, FG4)
	Food images	16	Make sure it’s good food photography and when you’re showcasing even from one question to the next, that it’s shot in a way that the portions don’t distort. I think that often we see—we certainly want real food images, so real food photography not drawn pictures and trying to make sure that the portion sizes are all in the same proportion as you move through the questionnaire. (Samantha, FG4)
Audio			
	General	5	I think it would be useful, even—I don’t know, but if you have people with visual difficulties as well then the sound would be good for that. (Rachel, FG6)
	Specific languages available	2	It speaks and you’ve got the pictures of the foods but all languages actually sound, audio and in the language of choice. (Fran, FG1)
Other features			
	Other ways to assess (non audio-visual)	7	Well personally I’ve always been in favour of the food frequency questionnaire approach at a macro level because it’s certainly can provide an opportunity for a ranking score that you can do just to give somebody some feedback on how they rank in terms of food choice. (Diane, FG5)
	Use language and concepts suitable to the audience	6	My experience with English as a second language, it’s not always the ESL * understand the word ‘serve’. In fact they can get confused, that they think a serve of fruit is a whole mango, or a whole rockmelon. So we use the word ‘portion’. (Sarah, FG3)
	Focus on foods not nutrients	5	Where you get your macronutrients from. It seems so basic to us but you do get people just unsure where their protein’s coming from or which foods are the ones that have the most effect on blood glucose. (Fran, FG1)
	Be explicit	3	The information we communicate is not actually helpful to people, because the other thing probably that I’d like to add, is that we spend a lot of time telling people why, why, why, why, justifying why, why, why. They don’t really care, they’re just tell me what to do. (Danielle, FG3)
	Provide feedback	2	If it’s going to be an online tool it’s got to be interactive with immediate feedback. (Diane, FG5)
	Be non-judgemental	1	So we need to get a much better pattern of what people are doing and to do that I think we have to do it in a very non-judgmental way or we won’t get the information. (Sonia, FG4)
	Use of examples	1	Well they’ve given all of the examples of food that would illustrate what they mean. (Fran, FG1)

^†,‡^ Names presented with the quotations are pseudonyms, and focus group number (FG1–FG6) is indicated. ^§^ There was significant cross-over with the thematic relationship of AGHE serves to everyday food presentation and single versus multi-ingredient or prepared foods. * ESL = English as a second language.
